# The Effect of Sanitizing Treatments on Respirator Filtration Performance

**DOI:** 10.3390/ijerph19020641

**Published:** 2022-01-06

**Authors:** Jürg A. Schütz, Anthony P. Pierlot, David L. J. Alexander

**Affiliations:** 1 CSIRO Manufacturing, 75 Pigdons Road, Waurn Ponds, Geelong, VIC 3216, Australia; tony.pierlot@csiro.au; 2 CSIRO Data61, 20 Research Way, Clayton, Melbourne, VIC 3168, Australia; david.alexander@data61.csiro.au

**Keywords:** respirator masks, COVID-19, sanitizing treatments, most penetrable particle size

## Abstract

The potential for alcoholic vapors emitted by common sanitizing treatments to deteriorate the (electrostatic) filtration performance of disposable respirator masks has been investigated. Reports in the literature and some standard test methods provide a confusing and ambiguous picture concerning the relevance of this effect. Four different types of exposure were investigated in this study to clarify the effect of alcoholic vapor emissions on respirator masks. These included exposure to saturated vapors, use of hand sanitizers, cleaning of table surfaces and sanitization of masks by spraying them with alcohol-containing solutions. Methods employed were designed to be as real-world oriented as possible while remaining reproducible. Filtration performance and deterioration effects on exposure to the different treatments were determined on three different types of certified commercial respirator masks—a P2 and two KN95 masks. This study provides substantial evidence that disposable respirator masks with an accepted performance rating are seriously compromised from an exposure to saturated alcoholic vapors, can tolerate a one-off spray treatment with an alcoholic solution and retain their attested protection under the influence of alcoholic vapors from the use of hand sanitizer or spray sanitizer. Considering the range of vastly different outcomes obtained from the four treatments investigated, it seems prudent to assess in each case the specific effects of alcoholic solution treatments and vapors on respirator masks before use.

## 1. Introduction

From early 2020, sanitizing treatments have fulfilled an essential role in suppressing the spread of infection during the COVID-19 pandemic [1]. Sanitizing is used in addition to other personal protective equipment (PPE) comprising gowns, face shields and face masks to protect people during essential activities ranging from working as a health care professional in hospitals to regularly visiting shops to purchase groceries.

The most common sanitizing treatments include alcoholic gel sanitizer for hand care and the spraying of alcoholic solutions onto table surfaces, door handles, keyboards, etc. to suppress the spread of viral loads via fomites, particularly where low ambient temperatures significantly slow the natural decay of coronaviruses [2,3]. Common products have a minimum of 60% alcohol content following recommendations by the Centre for Disease Control CDC [4] or even higher according to the World Health Organization WHO [5]. Ethanol is most commonly used for hand sanitizers in Australia because it is readily available, as well as isopropanol in some cases.

Another important tool in combatting the spread of disease is the use of face masks [6,7,8,9]. Having access to face masks is particularly important for hospital and aged care settings as well as crowded public areas (e.g., public transport, shopping centers, enclosed venues) where social distancing is difficult to observe or not practically feasible. Elastomeric respirators, i.e., masks equipped with cartridges or canisters, afford some of the highest levels of protection for health care workers [10] provided that effective and safe mask decontamination practices can be implemented. However, disposable respirators and surgical masks are often used [11], particularly if they are the only option available on the shelves in warehouses. The COVID-19 pandemic has led, predictably, to significant face mask supply shortages due to high demand world-wide. Finding replacements for face mask materials is important and particularly difficult because common alternatives do not provide the necessary high-level protection and acceptable breathability at the same time [12].

Another way to overcome supply shortages, which is often practiced in areas of significant disease outbreaks, is the reuse of disposable face mask after subjecting them to specialized treatments that, in essence, maintain the performance of the PPE [13,14,15,16] while deactivating the virus captured on the masks [17].

**Figure 1:**
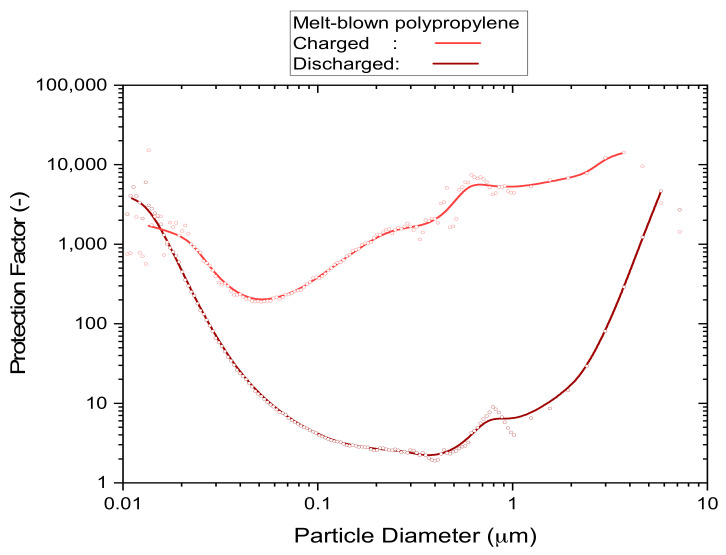
An example of the particle size dependence of the protection factor of uncharged and electrostatically charged filter media. Details of the P2 mask samples and method of test used to acquire the underlying data are described in Section 3.1.3.

Filter media that are either pleated or contain high levels of electrostatic charge achieve high protection levels from particles of all sizes. The reason for this is that at least one of these enhancement techniques is required to achieve an acceptable level of filtration performance under the typical constraints for face masks of size, weight and flow resistance. Disposable respirators, for instance, all work with electrostatic attraction to capture particles in a size range of approximately 0.05–2 µm, as shown in the example of Figure 1, which relates the particle concentration outside of the mask to the concentration that is breathed in (according to the definition of ‘protection factor’). These are the type of particles that are most difficult to capture because the normally predominant processes, which are based on fluid dynamics, are ineffective in this size range [18,19,20] (brown curve in Figure 1). It is the electrostatic field contained in these filter materials that captures aerosol particles almost exclusively in this sub-micrometer size range, rather than the physically visible filter material that acts as a screen. This is relevant because the actual size of coronaviruses is in the sub-micrometer size range, and simple activities such as breathing and talking alone already bear the potential to generate sub-micrometer aerosols from viral loads [21]. In terms of contracting disease, the viral load of inhaled sub-micrometer aerosols is relatively small, i.e., in comparison to aerosols and fomites created by body fluids or bacteria in coarser size fractions of several micrometers diameter [22]. However, the natural persistence of sub-micrometer aerosols, which keeps aerosol particles of this size suspended close to indefinitely [23,24,25], and a strong tendency to form larger agglomerates [26] as the aerosol ages over time can still lead to significant exposures in poorly ventilated areas [23,27], particularly if the viral strains are highly contagious [28,29,30,31].

It is also known that saturated alcohol vapors can significantly erode the electrostatic charge that is contained in filter media for the capture of sub-micrometer particles [14,15]. For instance, the use of electrostatically enhanced filter media is actively discouraged for use in air-conditioning systems by international standards such as the ‘high-efficiency particulate air’ (HEPA) filter standard ISO 29463 [32]. Air-conditioning standards generally recommend removing electrostatic charge from filter panels and products by exposing them for up to 24 h to saturated isopropanol vapors [33] or via immersion in isopropanol before measurement [34]. This approach is adopted because uncharged air-conditioning media are resistant to chemical (and biological [35]) challenges and can achieve the required filtration performance by a technique known as ‘pleating’. By folding the filter medium into pleats of several centimeters or several tens of centimeters depth it is possible to distribute the air flow through the panel across a much larger surface area than its natural cross-section. Provided there is sufficient space available, it is common to achieve 10-fold surface area increases relative to the cross-section of the panel, which reduces the speed of the airflow through the filter medium and increases dust holding capacity.

Pleating can also be used for canister- or cartridge-based respirators attached to elastomeric masks and these devices work in a similar way. However, disposable respirator products have emerged on world-wide markets in the last 20 years to seize a dominant role in aerosol exposure prevention, at least for low to medium demand applications, due to their low price and ease of mass production. All disposable masks use electrostatic charge rather than pleating for the purpose of achieving the required filtration performance mandated by standards such as the Australian Standard for respirators AS1716 [36] or the U.S. Standard for N95 masks that was developed by NIOSH [37,38].

Another issue that has been raised in the non-peer-reviewed pandemic literature, and which has been linked to the preference not to rely on electrostatically charged filter media in air-conditioning, is the question ‘Can alcoholic vapors adversely affect the electrostatic charge in filter media?’ [15,39]. The reason for this interest is the widespread use of alcohols (isopropanol or ethanol) for sanitizing treatments such as hand sanitizers for general hygiene or sanitizer sprays to clean fomite surfaces such as tables or door handles. 

The specific issue we investigated was the potential for alcohol vapors from hand sanitizers or other sources to render disposable masks ineffective or insufficiently effective for the intended purpose, which would apply to most surgical masks and disposable respirator masks that are offered commercially.

Masks were exposed to alcoholic vapors (ethanol or isopropanol) under four different exposure types: under (near) saturated conditions, from hand sanitization, from surface spray sanitization and from mask sanitization applied by spraying alcoholic solution directly onto the face of the mask. We demonstrate that the filtration performance of electrostatically charged disposable masks was substantially affected by alcoholic vapors near saturation but minimally affected by exposure to vapors generated from hand and surface sanitization.

## 2. Materials and Methods

### 2.1. Approach

The scope of this study was limited to investigating respirator masks and their capacity for respirable particle filtration, i.e., filtration involving sub-micrometer particles. Surgical masks were not included because they have an inherently lower (in-use) filtration efficiency due to the lack of a facial seal.

Since having an excellent facial seal is crucially important for the respirator mask to develop its full protective potential, it was deemed useful to mount the masks with a perfect facial seal. This was achieved by attaching the edge of the mask to a flat-plate using beeswax, which is a technique routinely used by NIOSH [40]. With this approach, it was possible to investigate potential deterioration of the filter medium without interference from external effects such as inward leakage or poor fit, while maintaining the respirator mask unchanged in its intended form.

Some of the tests were performed on flat-sheet samples that were cut from respirator masks.

### 2.2. Materials

#### 2.2.1. Face Masks

Common disposable respirator masks are made from a polypropylene multilayer composite that contains an electrostatically charged, melt-blown polypropylene filter layer. Masks of this type predominantly have a minimum particle collection (filtration) efficiency of 94% and come with a variety of different, but reasonably equivalent, performance requirements that are based on respirator standards, including those of Australia (P2 [36]), Europe (FFP2 [41]), United States of America (N95 [42]), China (KN95 [43]) or Korea (KF94).

The respirator masks used for this investigation were drawn from sources of P2-rated and KN95-rated products that were available in Australia from large retail or online hardware chains (Table 1) during a time of restricted supply. Masks certified under European Standards (FFP2) or South Korea (KF94) were not included due to limited availability. However, given that similar materials and production methods are used, the study should be relevant to a much wider range of certified masks.

Note the assignment of two different sample codes to the masks from Fujian Nuomigao, which originate from two different boxes of the same mask type. This was necessary because differences in physical properties between samples from different boxes were found to be significantly larger than those between samples from the same box.

Physical properties and filtration performances of these materials were measured and have been summarized in Table 2. Note that the typical ranges specified for the performance levels, comprising protection factor and quality factor, have been derived from 2–6 samples evaluated each from different batches and configurations (as whole masks or as flat-sheet sub-samples) of those masks.

Where parts of masks were tested as flat-sheets, the masks were first cut along the central seam to obtain flat media pieces. Discs of 109 mm diameter were subsequently marked on those pieces and cut out, followed by gluing rings of cardboard along the edges of the discs to obtain a 10 mm wide border that was perfectly flat and sealed. This procedure resulted in filter media with an active area of 89 mm diameter. The different sides of the mask were distinguished by a suffix ‘A’ or ‘B’ to investigate if different markings or embossments would make noticeable differences. Another reason was that strips of filter material that are used for automated mass production of respirator masks may exhibit performance differences between the left and right side of the strip. Since differences between ‘A’ and ‘B’ sides were generally small, the results from both sides were subsequently combined into an overall figure for the mask and reported as the relevant property.

Differences between different batches of the same type of mask were found for the KN95 mask manufactured by ‘Fujian Nuomigao’, where the thickness of the May batch mask material was found to be about 35% thicker than that of the April batch. There were also significant differences to other brands of KN95 masks (‘Zhejiang Shunfa’) or P2-rated masks (‘Shanghai Da Sheng’) according to results in Table 2. Standard deviations are shown in round brackets.

It was not part of this investigation to determine if respirator masks were fulfilling or failing the requirements of ratings claimed because the equipment used was of research grade and not set up for certification.

#### 2.2.2. Hand Sanitizer

Two types of antibacterial hand sanitizing gels were used:

- ‘Trafalgar’ by Brady Australia Pty Ltd., Greystanes, NSW, Australia, part no. 102606, containing 70% ethanol, 1 L dispenser
- ‘Germ Buster’ by Concept Laboratories Pty Ltd., Warana, QLD, Australia, code GB5LP, 70% ethanol/waterless, 5 L dispenser

**Figure 2:**
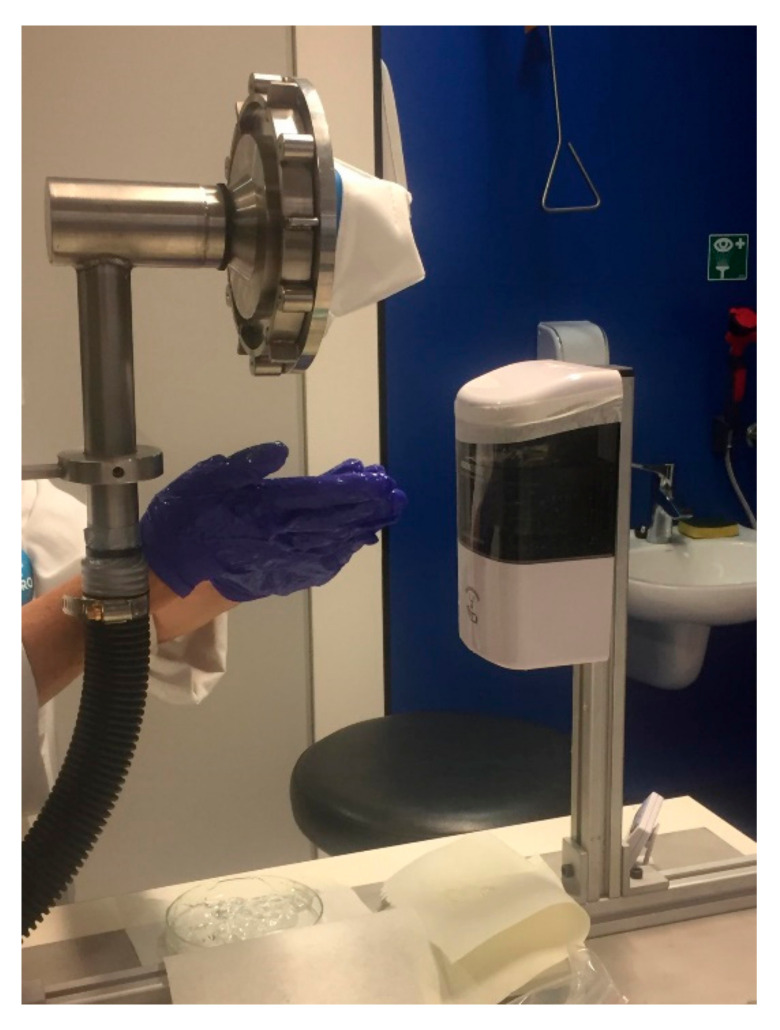
Experimental setup for exposing respirator test samples to alcoholic vapor (EtOH) emitted by an automatic hand sanitizer dispensing station via the release and use of gel hand sanitizer.

These gel sanitizers are compatible with the ‘automatic hand sanitizing dispenser’ (Figure 2) used in the experimental trials.

#### 2.2.3. Spray Sanitizing Solution

The spray sanitizing solution was made up from 70% analytical reagent grade propan-2-ol (Fisher Scientific, code P/7500/17) in reverse osmosis water.

#### 2.2.4. Other Chemicals

Aerosols were generated from 20 g/L analytical grade potassium chloride salt ‘Pronalys AR’ sold by Biolab Australia.

### 2.3. Methods

#### 2.3.1. Equipment and Protocols

The filtration performance of respirator masks and flat-sheet mask pieces (Table 1) was assessed via three different filter tests:

1. Flat-sheet screening test:

Flat-sheet mask pieces of 109 mm diameter were cut from respirator masks and subjected to non-destructive ‘screening tests’ (aerosol challenge of 0.01 mg/m^3^) using the equipment shown in Figure S1 of the Supplementary Information and also described elsewhere [44,45]. The filtration performance was generally assessed in a range of 0.3–0.5 µm particle size, although the instrument used (TSI 9306-03) provides information in 6 size channels reaching up to 25 µm particle diameter. A minimum of two sample pieces were characterized for each flat-sheet type and each exposure treatment.

This method established whether the filter media used in respirator masks maintained essential filtration performance under the influence of various challenges, including saturated alcohol vapors of ethanol or isopropanol.

2. Whole-mask screening test:

Whole respirator masks were mounted as received on a flat-surface adaptor by applying melted beeswax to the outer edge of the mask, using a procedure employed by NIOSH for N95 conformance testing [40]. The adaptor with mounted mask was filter tested in a modular set up, shown in Figure S2 of the Supplementary Information, that comprised a large stainless steel exposure chamber (15 L) with provisions to measure particle concentrations up- and down-stream of the mask as well as pressure drop. The aerosol challenge concentration was 0.01 mg/m^3^ and the detection range was 0.3–0.5 µm. Each measurement consisted of an average of 6–8 individual readings and a minimum of two mask samples were tested at each exposure level.

This non-destructive test method for whole masks was used to determine if an exposure to more ‘real-world’ sanitization treatments was likely to deteriorate the filtration performance of the masks.

3. Whole-mask MPPS test:

This method was used to characterize the penetration of a respirator mask as a function of particle size [18,19,32] in a range of 0.01–10 µm diameter and using a high aerosol challenge concentration of 10 mg/m^3^ that conforms to mask certification testing [36]. This required different detection systems that could operate at those particle concentrations (OPS TSI 3330 with 1:10 diluter TSI 3332-10) and were capable of extending the detection range down to 0.01 µm into the ultra-fine particle size range (SMPS Grimm 5.416). The test setup was similar to that used for ‘whole-mask screening’ but used an in-line test chamber (Figure S3, Supplementary Information [46]).

This method was used to determine the ‘most penetrable particle size’ (MPPS), which was the size of particles for which the filter medium of the respirator mask exhibited the poorest performance, hence, characterized the conditions where the mask was most vulnerable. This test method can change the filtration performance of a mask over the duration of the test due to the medium of the mask accumulating a large dust load that increases its pressure drop and changes penetration, with potential increases and decreases occurring with increasing load [47]. Results from masks tested in this work showed, typically, a 1% pressure drop increase over the duration of the test, while the effect on penetration remained insignificant.

All three test methods used a Digital Manometer (TSI, Dp-Calc model 5825) to measure the pressure drop at the set flowrate across the respirator mask or filter sample under test.

#### 2.3.2. Filtration Performance Indicators

The penetration P of a filter medium is calculated from measured particle concentrations, c_u_, in front of the filter test sample (up-stream) and concentration, c_d_, behind (down-stream) via Equation (1). The corresponding protection factor, S (suppression), is derived from P according to Equation (2) and the filtration efficiency, E, according to Equation (3)

P = c_d_/c_u_ (1)

S = 1/P = c_u_/c_d_ (2)

E = 1 − P = 1 − 1/S(3)

Another important performance indicator is the *breathing resistance*, as characterized in this work via the pressure drop, Δp. The pressure drop is the difference between the pressure in front of the filter test sample and the pressure behind the sample that is generated while a constant flow of air is passing through the filter test sample. Changing the flow rate of air through the filter test sample also changes pressure drop, so it is important to make comparisons under equivalent conditions.

The current Australian test standard for respirators [36] specifies inhalation resistance limits for two different exertion levels, which are specified by air flow rates, q_f_, through the mask of 30 L/min and 95 L/min, respectively. Note that respirator standards of other countries [41,42,43] may set different inhalation resistance limits, which needs to be accounted for accordingly.

Inhalation resistance also requires that the specified flow rate is applied to the whole respirator mask, or device, in its operational set up. For research purposes it is often more precise and economic to conduct filter tests on flat-sheet samples that are cut from specific parts of the respirator. Where flat-sheet filter samples are tested, it is therefore necessary to estimate the active surface area, A, of the mask and then calculate the face velocity, v_f_, according to Equation (4)

v_f_ = q_f_/A (4)

The flow rate through the flat-sheet filter sample is then adjusted to match the estimated face velocity of the whole mask in order to produce representative results. 

Another issue is the variability of physical properties such as the thickness or density of a filter medium. The effect of such properties can be effectively eliminated by working with the quality factor, Q, defined in Equation (5) [48]

Q = −ln(P)/Δp = ln(S)/Δp(5)

To illustrate the role of the quality factor, we consider the situation where two identical filter media with penetration P and pressure drop Δp are combined. The resulting medium has a reduced penetration of P^2^ and twice the pressure drop of 2·Δp, but quality factor Q remains unchanged.

A remaining dependence attached to Q is that of face velocity. This is passed on to the expression via the pressure drop. Its influence can be effectively eliminated via a modification based on Darcy’s Law, leading to the definition of quality factor, Q_x_, as in Equation (6) [44]

Q_x_ = Q·v_f_·η (6)

In this equation, η denotes the dynamic viscosity of air, which is 18.2 × 10^−6^ Pa·s at a temperature of 21 °C.

#### 2.3.3. Normalized Indicators

The deterioration of filtration performance was assessed for aerosol particles in the vicinity of the most penetrable particle size (MPPS) [18,19,20,32] in terms of normalized performance indicators described in Table 3. This normalization serves the purpose of mapping the effective level of charge stored in the filter medium (charge state) into a range where zero represents the completely discharged state and a value of 1 the fully charged state, hence, compensating for sample to sample variability.

The index ‘0.3′ stands for the particle size range of 0.3–0.5 µm diameter (Section 2.3.1). This provides the best representation of filtration performance near the MPPS that can be achieved by these instruments.

The ‘normalized’ performance indicators are based on the definitions of penetration P_0.3_ (Equation (1)) and quality factor Q_x0.3_ (Equation (6)) as evaluated for the 0.3–0.5 µm diameter particle size range. The definitions for ‘normalized’ filtration performance indicators are provided in Table 3.

The normalization of these parameters is such that a value of 1.0 indicates no change in filtration performance. However, realistically, due to statistical variability, this value can typically range between 0.9–1.1. These normalized parameters also do not go to zero when all or most of the electrostatic charge is lost because of a small contribution from direct physical particle capture that remains unaffected by the treatment.

### 2.4. Exposure to Alcoholic Vapors

#### 2.4.1. Exposure to Saturated Alcoholic Vapors

Alcoholic vapors were kept at saturation level in this setup by containing filter samples inside a desiccator, located on support grids above an amount of undiluted alcohol while being kept at a constant temperature of 25 °C inside an incubator. Flat-sheet media were suspended on three levels inside a 1.72-L desiccator with about 90 mL of liquid IPA alcohol at the bottom of the vessel. Whole-mask media were suspended one at a time in a larger 10-L desiccator with about 200 mL of liquid IPA alcohol at the bottom of the vessel.

The protocol for saturated alcohol exposure was relatively straight-forward with variation in exposure time being the main experimental parameter investigated. However, the investigation was slightly complicated by the fact that subsequent desorption of alcohol residues by means of forced ventilation for 30 min from a fume hood, post exposure, made significant differences to results. All samples were stored in plastic bags after exposure and after desorption. Samples were removed from their bags for flat-sheet screening testing, which subjected them to an air flow for approximately 10 min, and then returned to the bags for storage. The filtration performance as a function of storage time in plastic bags was monitored for 10 to 20 days in general, and up to 250 days in some cases where performance deterioration was strong.

The experimental parameters used for the alcohol vapor treatments and associated evaluations of filtration performance are summarized in Table 4.

#### 2.4.2. Hand Sanitizer Test Procedure

The protocols for the sanitizing treatments were an attempt to implement an exposure scenario that was typical of practice, yet severe enough to represent a worst case in those specific settings. The exposure time was the main experimental parameter varied, but other factors relating to the dispensing of sanitizing solutions needed consideration as well as means of control.

To simulate the effects of hand sanitization, a test was devised using a fan-blower to draw the vapors emitted from dispensing and using hand sanitizer gel through the respirator mask. The mask was mounted with beeswax on a flat surface with a circular opening underneath the mask, through which alcohol laden air from the vicinity of the dispensing station was drawn. Hand sanitizing activities were performed in the space between the dispensing station and the mounted mask (Figure 2).

The procedure involved dispensing EtOH gel hand sanitizer from an automatic dispensing station (VisionChart Healthcare, model HSDW-1) on an operator’s hands, rubbing of the hands for several seconds and waiting until the next scheduled gel release action occurred. Each release in the sequence contained an average of 0.67 g of gel sanitizer, with dispensing triggered by an optical sensor located at the bottom of the dispenser unit when a hand was held under the dispensing unit.

A series of preliminary experiments were conducted, as outlined in Table 5, to establish when a deterioration in filtration performance would occur. The schedule of 0.67 g individual gel releases was increased, starting from a sequence of 10 individual releases initially, and followed by periodic gel releases over the duration of 1 h, 2 h and 4 h to make up for the ‘total mass dispensed’.

Gel build-up on the gloves of the operator was periodically stripped off the gloves into a petri dish that was located underneath the mask.

On completion of the overall duration of the sanitizer release actions, the mask was removed together with its mount from the exposure setup and transferred to the ‘whole-mask screening’ filter test setup (Figure S2) to evaluate its filtration performance. The filtration performance was measured twice before treatment and twice after, where the first test of the treated mask was conducted immediately after treatment and the second test at least three days after treatment. Masks were stored in plastic bags between filter tests.

#### 2.4.3. Spray Sanitizing Test Procedure

Spray sanitizing exposure involved spraying an alcoholic solution containing 70% isopropanol by means of a trigger operated bottle atomizer onto a table surface, followed by manual wiping with a paper cloth for 10 s and waiting for the next, timed spraying action to occur.

**Figure 3:**
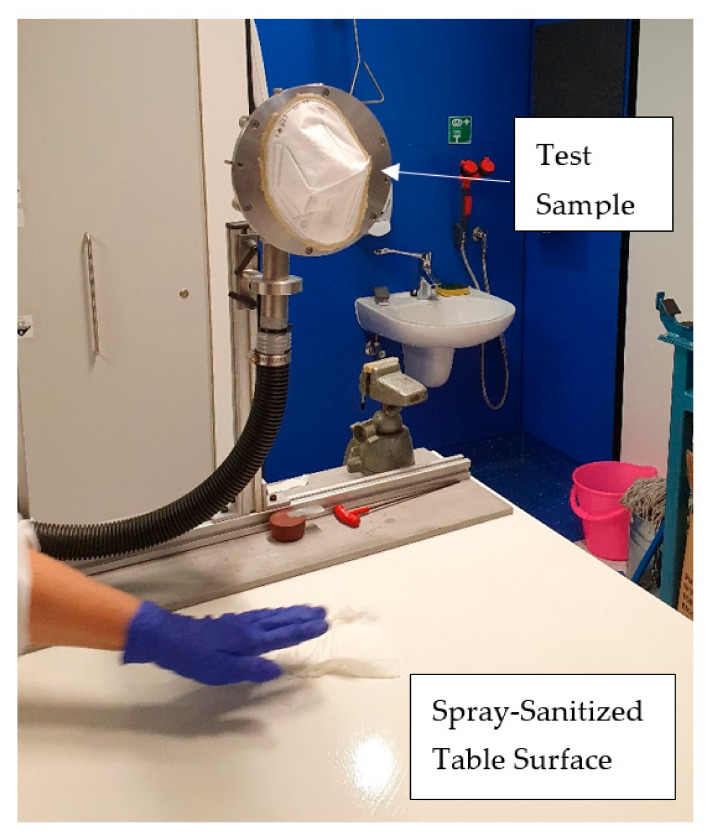
Experimental set up for exposing respirator test samples to alcoholic vapors (IPA) emitted by the process of spraying alcohol solution on a table surface followed by wiping with a paper towel.

Each spraying event of three pulls of the trigger released an average of 1.74 g of isopropanol solution. The mask was mounted with beeswax on a flat surface with a circular opening underneath the mask, through which alcohol laden air from above the treated table surface at 520 mm distance was drawn. Vapors emitted by the procedure were drawn through the respirator mask under test at a constant flow rate of 64 L/min (Figure 3).

Table 6 lists parameter settings used for this treatment. The setting of objectives for this exposure trial took guidance from experiences and outcomes of the hand sanitizer trial. The adopted exposure procedures followed a similar pattern to hand sanitizing, involving slightly different test parameters.

On completion of the overall duration of the spraying and wiping process, the mask was removed together with its mount from the exposure set up and transferred to the ‘whole-mask screening’ filter test setup (Figure S2) to evaluate its filtration performance. 

The filtration performance was measured twice before treatment and twice after, where the first test of the treated mask was conducted immediately after treatment and the second test at least three days after treatment. Masks were stored in plastic bags between filter tests.

During spray sanitization the accumulation of alcoholic vapors in the laboratory was sufficiently high that it was deemed necessary for staff to don a chemical vapor respirator for protection.

#### 2.4.4. Mask Sanitization Test Procedure

This procedure addressed a potential scenario where a person might decide to use alcoholic cleaning solution to disinfect a worn mask for continued use.

Alcoholic solution containing 70% isopropanol was sprayed directly onto the mask from a distance of 150 mm to 200 mm by means of a trigger operated bottle atomizer. Each spraying event of three pulls of the trigger released an average of 1.74 g of isopropanol solution. The mask was subsequently dried for 1 h under the bell of a snorkel extractor.

The filtration performance was measured twice before treatment and twice after, where the first test of the treated mask was conducted immediately after treatment and the second test at least three days after treatment, with storage in plastic bags between tests.

## 3. Results

### 3.1. Exposure to Saturated Alcohol Vapor

The ‘worst-case’ screening test was designed to measure the loss of filtration performance from a static exposure to an atmosphere saturated with alcoholic vapor, as illustrated by the brown curve (discharged) of the example shown in Figure 1. It is a method that is commonly used by air-conditioning filter standards [33] to remove electrostatic charge from filter media prior to the actual performance test. When early test results (not shown) revealed severe deterioration in filtration performance after 24 h of exposure to saturated isopropanol vapor due to the depletion of electrostatic charge, a more detailed investigation at lower exposure times (5, 1 and 0.1 h) was undertaken.

Respirator masks with P2- and KN95-ratings (Table 1) were tested and normalized performance indicators defined in Table 3 were determined. A normalized value of 1.0 indicates that the filtration performance remained unaffected by the treatment, while a value of 0.0 indicates that the filter material completely lost its barrier properties.

Results from exploratory experiments are provided in the Supplementary Information S2.

#### 3.1.1. Mask to Mask Variability

Physical properties listed in Table 2 show that differences between disposable masks from different batches (KN95-FN1 versus KN95-FN2), or even between different boxes from the same batch (KN95-FN1, Box 1 versus Box 2), can be significant. The variability in filtration performance was determined via ‘whole-mask screening’ tests conducted on five different masks from the same box within a batch of the P2-SD masks.

The masks were exposed for 5 h to saturated IPA vapors, followed by 30 min of ventilation in a fume hood. The masks remained mounted during storage in plastic bags for additional non-destructive whole-mask screening tests.

**Figure 4:**
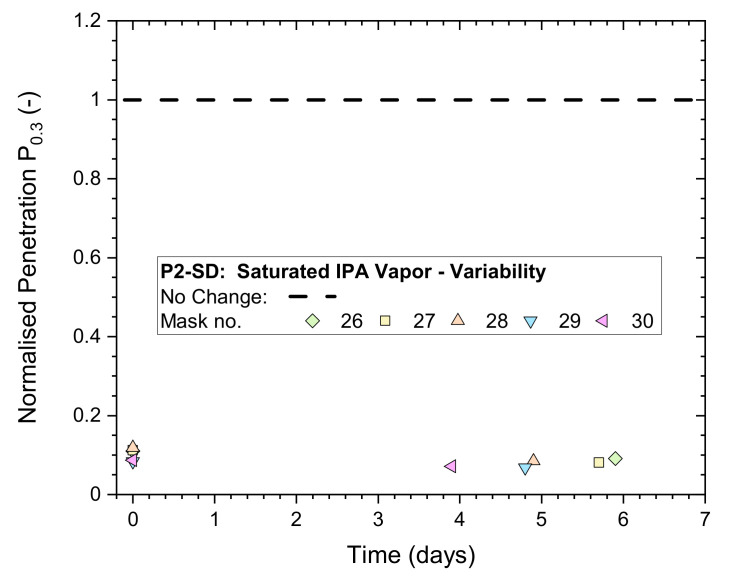
Normalized penetration P_n0.3_ from whole-mask screening tests of a P2-rated mask (P2-SD: Shanghai Da Sheng) after exposure to 5 h of saturated IPA vapor followed by 30 min of ventilation in a fume hood, measured immediately after treatment and after storage in plastic bags for up to 6 days.

Results for normalized penetration P_n0.3_ measured immediately after treatment and once more within the following 6 days are plotted in Figure 4. All P_n0.3_ results were in the vicinity of 0.1, clearly indicating that mask performances were heavily affected by the exposure.

#### 3.1.2. Differences between Makes

**Figure 5:**
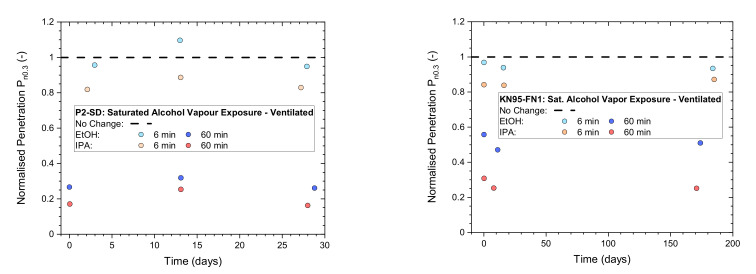
Normalized penetration P_n0.3_ from flat-sheet screening tests of a P2-rated mask (**left**: Shanghai Da Sheng, P2-SD) and a KN95-rated mask (**right**: Fujian Nuomigao, KN-95-FN1) exposed to saturated alcohol vapor at low (6 min) and medium (60 min) levels after ventilation for 30 min in a fume hood and then testing after storage in plastic bags for up to 200 days.

Low and medium exposure times of 6 and 60 min were used to compare (Figure 5) the performance of P2-rated masks (Shanghai Da Sheng) to a KN95-rated mask (Fujian Nuomigao). After exposure to saturated IPA vapor, the masks were ventilated by a fume hood for 30 min to assist with desorption of vapors. All samples were stored in plastic bags after ventilation and between testing up to 200 days after exposure.

Results of the two masks are overall quite similar and remain stable over time. This is not unexpected due to strong similarities in the construction of these respirators, containing an electrostatically charged, melt-blown filter layer made from polypropylene.

However, overall filtration performances listed in Table 1 show that the P2-rated mask has a lower breathing resistance with an average pressure drop of 210 Pa (at v_f_ = 0.15 m/s) compared to 220–290 Pa for the KN95 masks.

#### 3.1.3. Particle Size Dependence

The detrimental effects of saturated IPA vapors on filtration performance are not the same for all particles but vary in non-linear fashion as a function of particle size. ‘Whole-mask MPPS’ testing with a high aerosol particle concentration challenge (10 mg/m^3^) was used to characterize this dependence using five respirator masks exposed to 5 h of saturated alcohol vapor and ventilated for 30 min in a fume hood. Prior to MPPS testing, the mask samples were tested non-destructively by ‘whole-mask screening’ tests (described in Section 3.1.1) to validate the integrity of the mask’s border seal. Mask samples were stored between tests, while mounted, in plastic bags.

**Figure 6:**
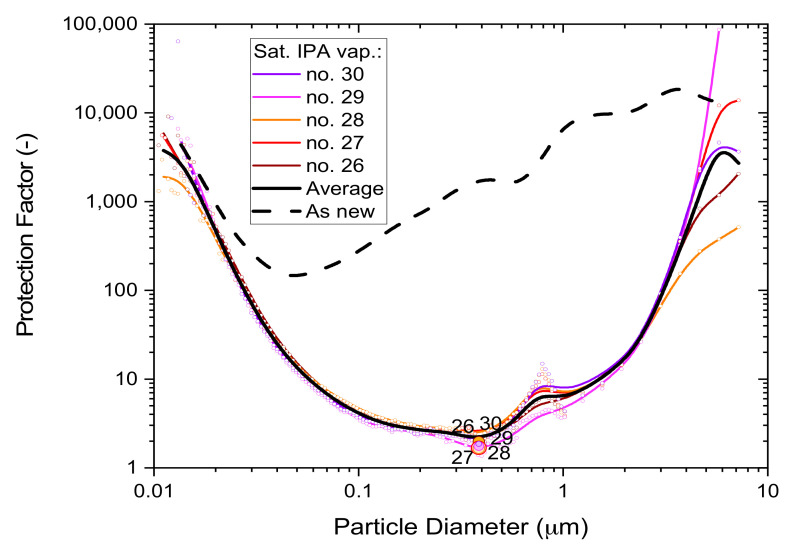
The particle size dependence of the protection factor of five P2-SD disposable respirator masks (Shanghai Da Sheng, box 3) after 5-h exposure to saturated IPA vapors; results from non-destructive testing are shown as circular dots with corresponding coloring at 0.39 µm particle size. The average performance is shown by the black solid line and the performance of an unused mask (as new) is shown for comparison by the black dashed line.

The results in Figure 6 show individual performances of the masks, the average dependence from all five masks (black solid line) and the typical performance of an unused mask (black dashed line) for comparison. The minima of these curves represent the conditions where the masks are most vulnerable, as characterized by the ‘Most Penetrable Particle Size’ MPPS.

The filtration performance difference between exposed masks and unused masks is quite large and shows the detrimental effect of saturated alcohol fumes on disposable respirator masks. Since saturated alcohol vapors deplete electrostatic charge, these results also illustrate the important role that electrostatic enhancement plays in relation to melt-blown polypropylene filter media.

### 3.2. Exposure to Hand Sanitizer Vapor

The results above demonstrate that the performance of masks utilizing electrostatic charging can degrade significantly when exposed to high concentrations of alcoholic vapors. Thus, it is important to identify the extent of performance degradation that may occur when disposable respirators are used in ‘real-world’ settings where exposure to alcoholic vapors is almost certain.

Hand sanitizer dispensing stations are currently used in many places, ranging across shopping centers, restaurants, medical practices, aged care homes, hospitals and more. The concentration and persistence of alcohol vapor emitted by dispensing stations can vary and will depend on factors such as the frequency and number of release actions, the level of air movement and temperature. The procedure adopted was between realistic ‘real-world’ and ‘worst case’ exposure levels. If the masks were found to function, then the tested exposure conditions provided assurance, at a high level of confidence, that the devices should perform as desired.

Non-destructive whole-mask screening tests were used to validate the filtration performance of exposed respirator masks to prolonged exposure times to vapors generated from numerous hand sanitization actions.

**Figure 7:**
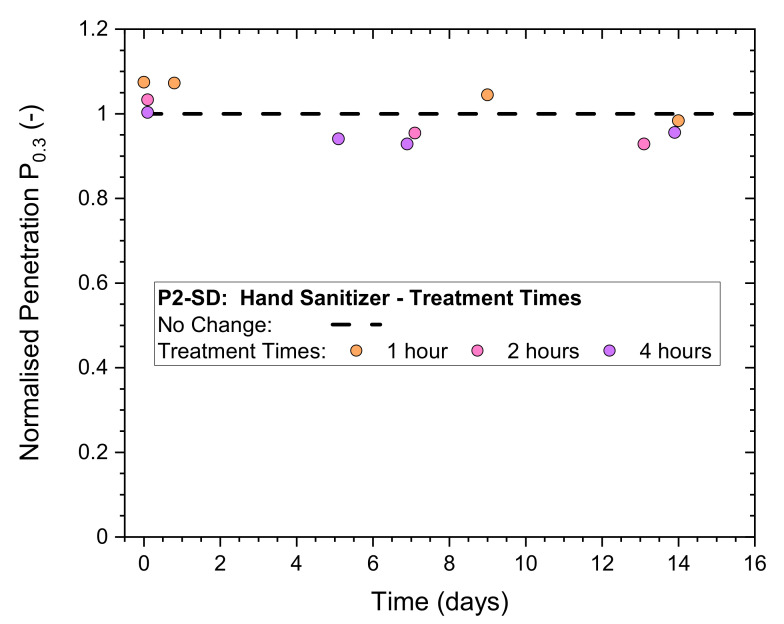
Normalized penetration P_n0.3_ from whole-mask screening tests of a P2-rated mask (P2-SD) after exposure to numerous (continuous) hand sanitizing actions for different periods of time, measured immediately after treatment and after storage in plastic bags for up to 14 days.

Results for normalized penetration P_n0.3_ for exposure levels 2–4 in Table 5 have been plotted in Figure 7. The initial P_n0.3_ at time zero are in a range of 1.0–1.1 and remain close together for the subsequent storage period (in plastic bags) monitored for 2 weeks. This result is a clear indication that the masks remained unaffected for treatment times up to 4 h.

**Figure 8:**
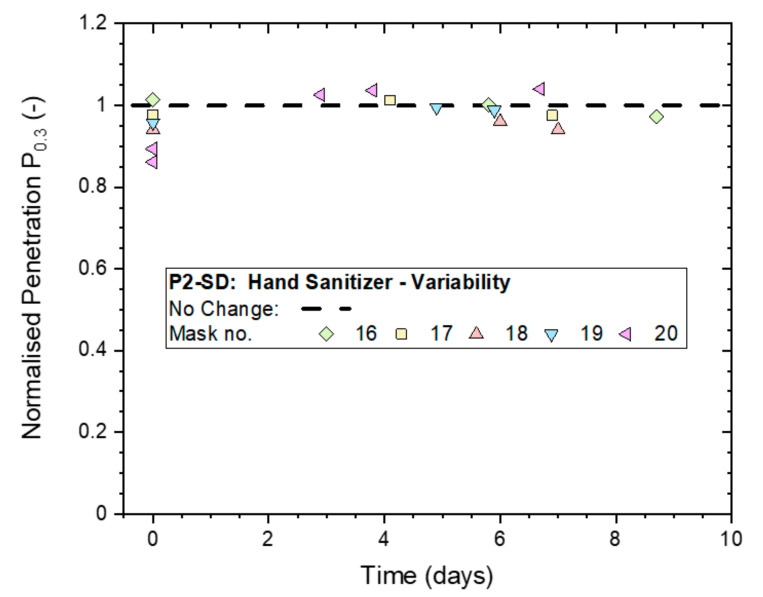
Variation in the normalized penetration P_n0.3_ from whole-mask screening tests between five masks (P2-SD: Shanghai Da Sheng) after exposure to continuous hand sanitizing actions for 4 h and subsequent storage in plastic bags for up to 9 days.

The 4-h treatments and testing were subsequently repeated on five different samples of the P2-rated masks to establish the typical variability of the effect of hand sanitizer exposure. The results in Figure 8 illustrate that all masks remained unaffected except for Mask ‘20′, which dropped initially to a P_n0.3_ just below 0.9 but subsequently recovered to 1.0, which was its original, untreated filtration performance. It must be noted that the (absolute) penetration remained below 2% at every stage of testing for all five masks.

**Figure 9:**
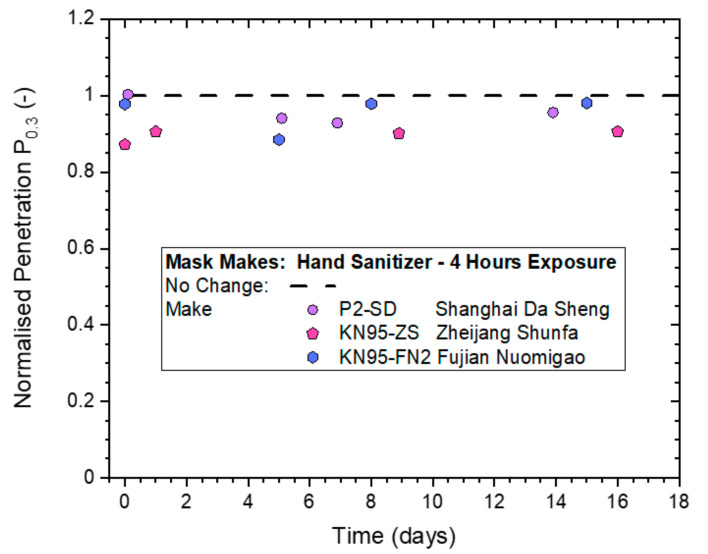
Normalized penetration P_n0.3_ from whole-mask screening testing of three different makes of disposable masks exposed to continuous hand sanitizing actions for 4 h (exposure level 4), measured immediately after treatment and then after storage in plastic bags for up to 16 days.

Since no significant deterioration in filtration performance from non-destructive ‘whole-mask screening’ testing was detected, even for the highest treatment level that involved 4 h of continuous exposure, it was used to compare the performances of the two different KN95-rated masks to the P2-rated mask from Shanghai Da Sheng. The results in Figure 9 indicate that all three masks gave P_n0.3_-values in the range of 0.9–1.0, which suggests that deviations from the ‘No Change’ value of 1.0 are all within statistical variability of the method. This led to the conclusion that all three masks remained effective after a 4-h long exposure to hand sanitizer vapors.

Respirator masks exposed to four hours of hand sanitization were finally subjected to a ‘whole-mask MPPS’ test with a high aerosol particle concentration challenge (10 mg/m^3^) to establish filtration performance under conditions where the masks are most vulnerable. This is the ‘Most Penetrable Particle Size’ MPPS. The mask samples were tested non-destructively by ‘whole-mask screening’ tests, prior to MPPS testing, to validate the integrity of the mask’s border seal, and mask samples, while mounted, were stored between tests in plastic bags.

**Figure 10:**
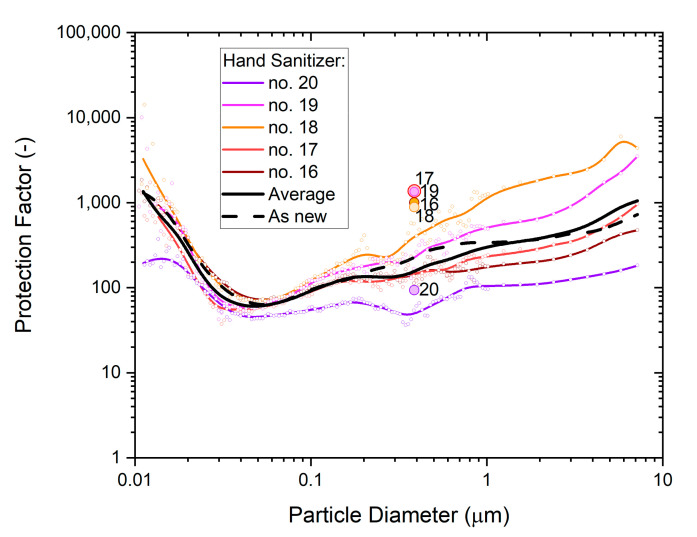
The particle size dependence of the protection factor of five P2-SD disposable respirator masks (Shanghai Da Sheng, box 2) after exposure to hand sanitizer vapors; colored dots represent particle size-specific measurements (SMPS, OPS) of the respective mask, with b-spline curves providing visual context; results from non-destructive testing are shown as circular dots with corresponding coloring at 0.39 µm particle size. The geometric average of the five masks is represented by the black solid line, and the performance of a single unused mask (as new) is shown for comparison by the black dashed line.

The statistical variability of the five P2-SD masks from ‘whole-mask MPPS’ testing is illustrated in Figure 10. The average across the five masks is shown by the solid black line, while the performance for untreated is shown ‘as new’ by the dashed black line.

Results show a clear spread between measurements, but the overall trends are similar for all masks. The non-destructive tests produced overall slightly higher protection factors than particle size dependence tests showed at the same particle size. This variance is in part due to measurement uncertainty and in part due to test method differences.

### 3.3. Exposure to Spray Sanitizing

Experiments of this trial were concerned with the common hygiene practice of spraying alcoholic solutions on table surfaces, door handles and controls of appliances to remove potential bacterial and viral fomites to curb the spread of infections.

**Figure 11:**
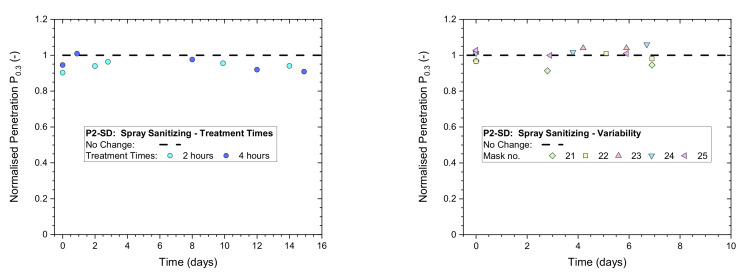
Normalized penetration P_n0.3_ from whole-mask screening testing of a P2-rated mask (Shanghai Da Sheng) exposed to continuous spray sanitizing actions (**left**) for 2 or 4 h; (**right**) variability between 5 masks from the same batch and box exposed to 4 h of continuous spray sanitizing actions.

The effect of exposure time (2 and 4 h) on a disposable P2-SD respirator from continuous spray sanitizing treatments and the mask-to-mask variability obtained from five P2-SD masks exposed for 4 h are illustrated in Figure 11. All normalized penetration measurements from whole-mask screening tests conducted for these exposures remained above 0.9, which indicates the masks maintained a high protection factor immediately after exposure as well as after storage in plastic bags for up to two weeks. Samples were removed from their bags for filter testing. This subjected them to an air flow for approximately 15 min before they were returned to the bags for storage.

**Figure 12:**
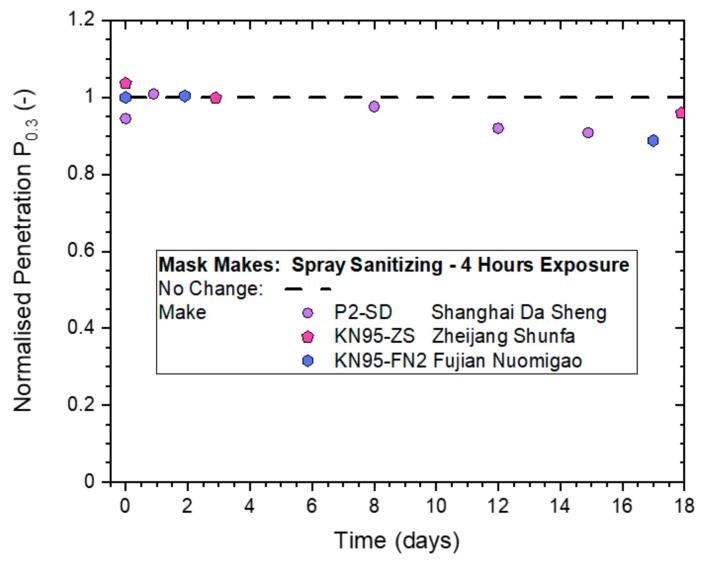
Normalized penetration P_n0.3_ from whole-mask screening testing of three different makes of disposable masks exposed to continuous spray sanitizing actions for 4 h.

The effect of 4-h continuous spray sanitizing treatments on the two KN95-rated respirators (KN95-ZS, KN95-FN2) is compared in Figure 12 to results from a P2-SD respirator. All normalized penetration measurements from whole-mask screening tests conducted on the masks remained above 0.85, which indicated that differences between those different makes of masks were small.

**Figure 13:**
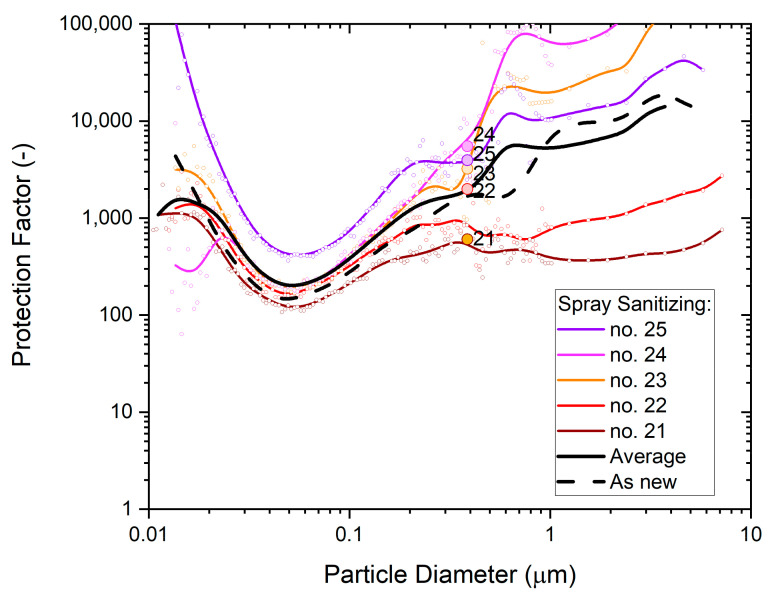
The particle size dependence of the protection factor of five P2-SD disposable respirator masks (Shanghai Da Sheng, box 3) after exposure to vapors from spray sanitizing; results from non-destructive testing are shown as circular dots with corresponding coloring at 0.39 µm particle size. The average performance is shown by the black solid line and the performance of an unused mask (as new) is shown for comparison by the black dashed line.

A new set of five P2-SD masks (Box 3) were exposed to continuous spray sanitizing for 4 h, ventilated for 30 min and subjected to whole-mask MPPS testing. The results in Figure 13 illustrate the statistical variability in filtration performances from different masks.

The average protection factor dependence of the five masks is shown as the black solid line. It is very close to the typical performance of untreated masks from the same box, which is shown as the black dashed line. This supports the conclusion drawn from whole-mask screening tests that an exposure of the masks to spray sanitizing has no significant effect on filtration performance.

Note the significant difference between MPPS screening tests in Figure 10 and Figure 13, which were obtained from P2-SD masks sampled from different boxes. The physical properties in Table 2 showed significant differences for masks from two different batches, KN95-FN1 and KN95-FN2, manufactured by Fujian Nuomigao. Results from the MPPS screening tests indicate that significant differences can also occur for different boxes from the same batch of masks. This source of error was controlled by measuring untreated mask samples from the same box and batch for all comparisons of filter tests made and for all types of filter tests used.

### 3.4. Exposure to Mask Sanitization

The ‘mask sanitization’ scenario was motivated by reports of disposable masks being reused after spraying the outer surface with an alcoholic solution to kill any germs accumulated during use or to make them more agreeable to wearing by spraying with perfume. Experiments devised to address these scenarios involved spraying a table cleaning solution with 70% IPA content directly onto the masks (three atomizer trigger actions from three different directions) and ventilating the masks for 1 h for drying. Multiple treatments of this type were not explored as the reuse of disposable masks is not recommended by manufacturers. In practice, these types of treatments would also be highly variable (e.g., amount applied, wicking and absorbance characteristics of mask surface and interior layers) making it difficult to devise an experimental approach and analysis without ambiguity in interpretation.

**Figure 14:**
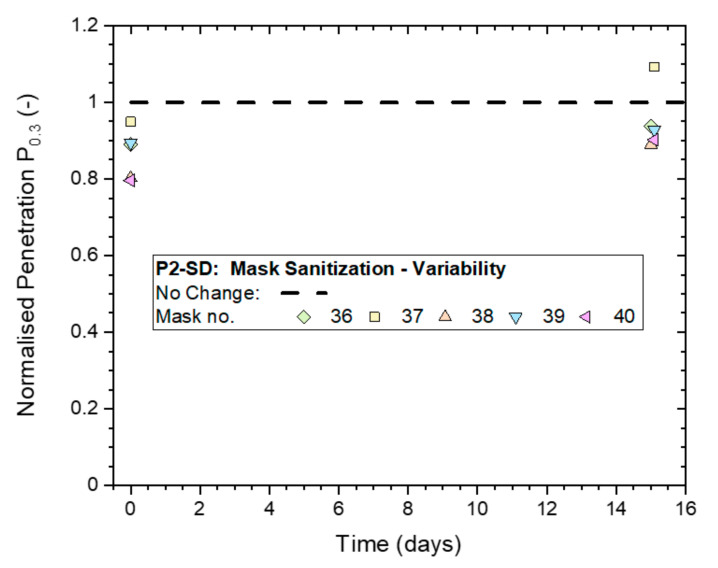
Variation in the Normalized penetration P_n0.3_ from whole-mask screening tests between five masks (P2-SD: Shanghai Da Sheng) after exposure to mask sanitization application of 70% IPA solution and subsequent storage in plastic bags for up to two weeks.

The effect on filtration performance was assessed by ‘whole-mask screening’ tests, with two tests conducted before mask sanitization exposure, one test immediately after exposure and a follow-up test after two weeks. Masks were stored in plastic bags between tests. Normalized penetrations P_n0.3_ from these tests are shown in Figure 14.

Results measured immediately after treatment were in a range of 0.8 to 1.0, and increased to 0.9 to 1.1 for the follow-up tests conducted two weeks later. This indicates that the masks might have temporarily experienced a slight drop in filtration performance that was recovered in the two weeks that followed.

**Figure 15:**
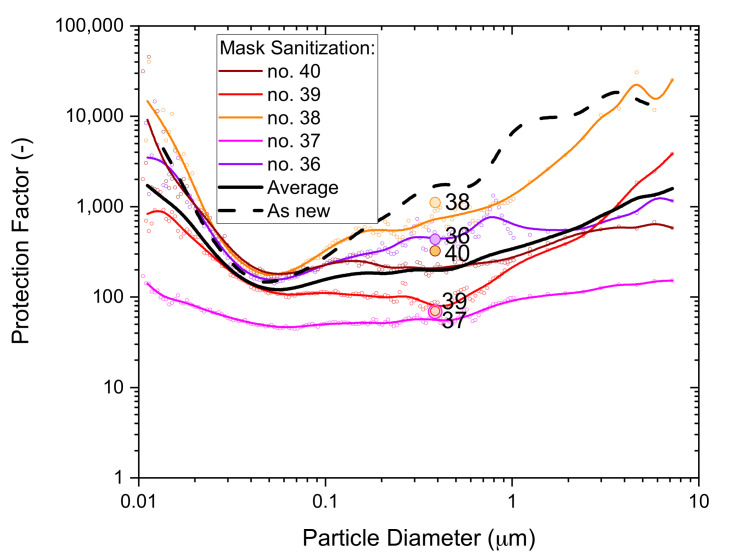
The particle size dependence of the protection factor of five P2-SD disposable respirator masks (Shanghai Da Sheng, box 3) after exposure to mask sanitization application of 70% IPA solution; results from non-destructive testing are shown as circular dots with corresponding coloring at 0.39 µm particle size. The average performance is shown by the black solid line and the performance of an unused mask (as new) is shown for comparison by the black dashed line.

These five masks were subjected to ‘whole-mask MPPS’ testing to obtain additional information, comprising particle size dependence and performance under elevated strain from using a higher particle concentration. These results are shown in Figure 15. 

Comparing the average from the five masks tested (black solid line) to the typical performance of an unused mask (black dashed line) supports the conclusion that the filtration performance of some masks, at least, may have deteriorated as a result of the mask sanitization treatment. The deterioration from three spray actions was likely only small, but using more extensive spray treatments might lead to significant filtration performance deteriorations.

### 3.5. Statistical Analysis of Results

A statistical analysis of the ‘whole-mask screening’ test results shown in Figure 4, Figure 8, Figure 11 and Figure 14 was undertaken to determine if results from the treated samples of the four experimental series were statistically different from untreated samples.

#### 3.5.1. Statistical Model

The primary observation variable was the measured absolute penetration of whole-mask samples (as opposed to the ‘normalized penetration’ shown in the plots above) and differences between the untreated and treated states after exposure to alcoholic challenges. Secondary observation variables were pressure drop, sample weight and time between treatment and measurement.

Mixed models for the logarithm of penetration were fitted with the lme4 package in R [49] and compared using analysis of variance to test the effects of the various treatments. Calculated *p* values less than 0.05 are considered statistically significant when comparing models with or without certain parameters. Confidence intervals for penetration were calculated using bootstrapping.

#### 3.5.2. Variability of Untreated Mask Samples

**Figure 16:**
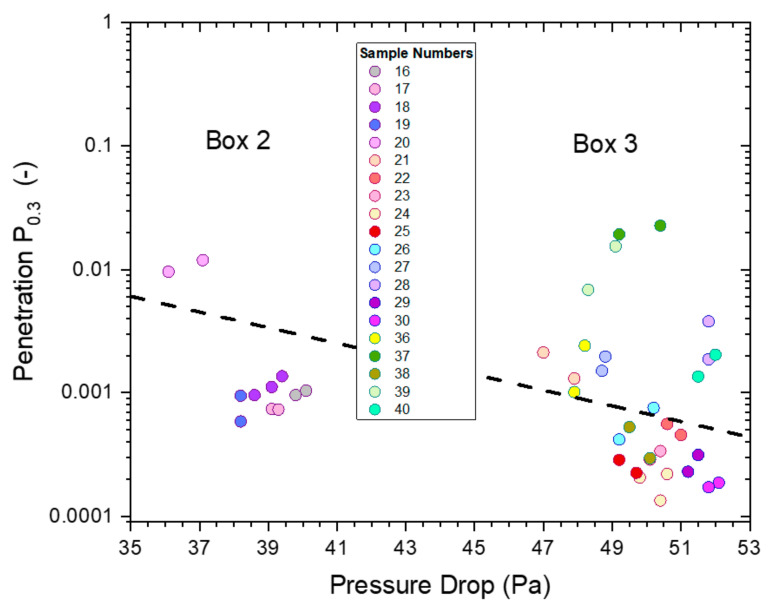
The penetrations from ‘whole-mask screening’ tests of untreated mask samples (P2-SD) for particles of 0.3–0.5 µm diameter as a function of pressure drop. The different colors represent different mask samples with repeat measurements shown in matching colors.

The (absolute) penetration of all untreated masks (P2-SD) as a function of pressure drop for particles of 0.3–0.5 µm diameter is shown in Figure 16. All measurements were conducted on P2-SD mask samples, with those used for the hand sanitizer trial coming from box 2 (Figure 8) and those used for saturated vapors (Figure 4), spray sanitizing (Figure 11) and mask sanitization (Figure 14) taken from box 3.

The penetration was below 3% for all masks with the pressure drop falling into two groups of similar pressure drop, which were linked to the specific box the mask samples came from: box 2 had a pressure drop between 36–40 Pa, while the range for samples from box 3 was 47–52 Pa.

The relationship between pressure drop and the logarithm of penetration, Ln(P), is linear, according to Darcy’s Law, which is depicted by a dashed black line. This relationship is useful to illustrate the expected change in penetration and pressure drop in the range of mask samples used from boxes 2 and 3. This relationship assumes that the quality factor Q (and Q_x_) for the filter media is the same for each box, which is not unreasonable as all masks were likely to be made from the same materials with slight differences in weights and packing density giving rise to the observed changes in penetration and pressure drop.

**Figure 17:**
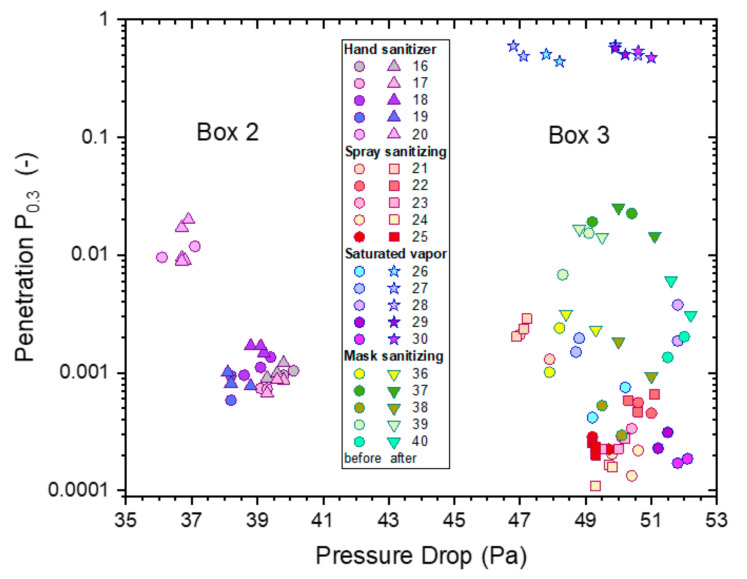
The penetrations from ‘whole-mask screening’ tests from treated and untreated mask samples for particles of 0.3–0.5 µm diameter as a function of pressure drop for each sample before and after four different alcoholic exposure treatments: saturated vapor treatments (5 h, IPA, stars), hand sanitizer (4 h, EtOH, triangles), spray sanitizer (4 h, IPA, squares) and mask sanitization (3 spray pulls, IPA, inverted triangles). For comparison purposes, the results of samples before treatment (Figure 16) are also included, represented by circular symbols with the same coloring as the respective treated samples.

The effect of the four exposure treatments is presented in Figure 17 as paired comparisons for each mask. All mask samples used within an exposure treatment were selected from the same box. A comparison of results from untreated masks out of boxes 2 and 3 (Figure 16) suggests that the main distinguishing factors were differences in pressure drop (and penetration). The effects of treatments were assessed based on before and after measurements on the same mask, avoiding confounding with mask differences.

## 4. Discussion

We conducted several exploratory investigations concerning exposure to saturated IPA vapors and ventilation post-treatments (Supplementary Information S2.1). By exposing sets of five masks to four different alcoholic exposure treatments, we determined the effects of each of the treatments on the filtration performance of disposable respirators. The magnitude and statistical significance of effects caused by the treatments were assessed by statistical analysis of the differences in filtration performances of the five masks before and after treatment. Since the ‘whole-mask screening’ tests used were non-destructive tests, it was possible to subject the same masks to ‘whole-mask MPPS’ tests to determine the particle size dependence of the protection factor.

### 4.1. Different Exposure Treatments

Figure 17 presents filtration screening test data from four different treatments, and this was analyzed statistically by creating models for pressure drop and penetration. Masks still have excellent filtration efficiency after hand or spray sanitizer and mask sanitization treatments. However, masks have uniformly high penetration (poor filtration efficiency) after saturated IPA vapor treatment.

A mixed model was fitted to the data shown in Figure 17, incorporating random variation between masks as well as between individual measurements. A logarithmic scale is appropriate since penetration is calculated as a ratio of two concentrations.

The clearest observation in Figure 17 is the extremely large effect of 5 h saturated IPA vapor; all masks with this treatment have absolute penetrations between 0.44 and 0.61, and no other measurements exceed 0.2. These masks had similar penetration after treatment despite widely varying penetration levels before treatment; variation between masks was thus modelled to be much lower after this treatment. A similar effect applies, to a lesser degree, for the mask sanitization treatment.

The IPA vapor treatment (5 h) was modelled to increase penetration P_0.3_ by a factor in excess of 500, which is obviously statistically significant (*p* < 10^−9^), and the mask sanitization treatment was modelled to increase penetration by a factor of 3, also statistically significant (*p* < 10^−3^). Factoring in these treatments and their effects on variation between masks, the effect of time since treatment was also statistically significant (*p* = 0.02); in this model, hand sanitizer treatment also had a statistically significant effect on penetration (*p* = 0.008), increasing penetration by 29% on average. Penetration was modelled to decrease by 2.3% each day after treatment.

Statistical tests indicate no other significant effects (including the sample or basis weight) on the combined data.

Even statistically significant effects may not have any real functional significance; thus, Table 7 provides the expected penetration levels P_0.3_ before treatment and immediately after each of the four treatments, according to the statistical model. Saturated IPA vapor treatment is expected to lead to very high penetration. Hand sanitizer and mask sanitization treatments are modelled to increase penetration, though the effect size is low enough that the average difference in penetration would not be of any practical importance.

One-sided 95% confidence intervals for penetration P_0.3_ of a randomly selected mask before and immediately after the various treatments are also listed in Table 7. The penetration levels after hand sanitizer, spray sanitizer and direct spray mask sanitization treatments can confidently be concluded to be well below regulatory values, but the penetration level after saturated IPA vapor treatment can confidently be concluded to be well over the regulatory value. The confidence intervals are for an individual measurement, allowing for variation between masks and between measurements. They were calculated from 10,001 bootstrap samples.

### 4.2. Future Research

While results from this study provide insights into conditions where electrostatic performances of common disposable respirators are affected or remain unaffected by exposure to alcoholic vapors, there are further aspects of common use that were not covered in this work. These include: the combined influences of the humidity of exhaled air and aerosolized body fluids, changes in temperature, non-constant airflow in a typical breathing cycle, and breathing volume and rates. The most representative approach towards accounting for such influences would involve running trials in actual work settings. Such trials do, however, have the disadvantage that they are difficult to control and that the complex network of influences is difficult to break down into individual factors.

Nevertheless, while the disposable respirator masks failed under the most extreme case of exposure, the masks performed very well in the more experience-based exposure settings. In particular, in view of the considerable rigor that was applied by the two experience-based challenges, the fact that the disposable respirators retained excellent protection performance should instill confidence that these devices can indeed provide reliable protection for the purposes for which they were made.

## 5. Conclusions

The effects of alcoholic vapors (ethanol and isopropanol) released during use of hand sanitizers or alcoholic sprays on the protective properties of common disposable respirator masks has been investigated.

The study looked at different types of challenges at three different severity levels. 

The most severe challenge involved exposing the masks to saturated alcoholic vapors, similar to the conditioning methods [33] typically used for the certification of air-conditioning products. Results revealed a large deterioration in particulate protection for exposures of 60 min or longer, with the deterioration easing to insignificant as the exposure period was reduced to 6 min.

The other three challenge types were designed to represent more real-world exposure scenarios, which people might experience when wearing respirator masks indoors in hospitals, office spaces, shopping centers and the like. The chosen experimental settings included ‘worst-case’ scenarios based on an ‘experience’ level that may be encountered during attempts to mitigate exposure to the SARS-CoV-2 virus:

1. The use of gel hand sanitizer, as commonly provided in many places by automatic or manual dispenser units, to maintain good personal hygiene;
2. The spraying of 70% isopropanol solution on table surfaces for general hygiene purposes and for the removal of fomites;
3. Sanitizing respirators by spraying 70% isopropanol solution directly onto the face of the mask.

Results from heavy exposures over 4 h showed respirator masks exposed to those types of alcohol vapors retained excellent protection performance in all three of these scenarios, i.e., where alcoholic vapors are present in the vicinity of hand sanitizer dispenser units, during the cleaning of table surfaces or after masks are sanitized with small amounts of 70% isopropanol solution.

## Figures and Tables

**Table 1:** Filter material description and references.

Filter Mask	Manufacturer	Retail	Type	Manufacturing Date	Rating	Sample Reference
Respirator with ear loops	Fujian Nuomigao Medical Technology Co., Ltd., Jinjiang, Fujian, China	Bunnings	SOJO DR20V (Box 1)	13 April 2020	KN95	KN95-FN1
			(Box 2)	4 May 2020	KN95	KN95-FN2
Respirator with ear loops	Zhejiang Shunfa Safety Technology Co., Ltd., Jinhua City, Zhejiang, China 321200	Seton	SF-K01	July 2020	KN95	KN95-ZS
Respirator mask, elastic band straps	Shanghai Da Sheng Health Products Co., Ltd., Songjiang, Shanghai, China 201613	Bunnings (Protector)	DTC3B P2 (RP2FR20)	February 2020	P2	P2-SD

**Table 2:** Filter material specifications, and properties (standard deviations in brackets).

Sample Reference		Thickness	Basis Wt.	Packing Density	Pressure Drop	Protection Factor (0.3)	Quality Factor (0.3)
		(mm)	(g/m^2^)	(%)	(Pa)	(−)	(10^−9^ m)
KN95-FN1	Overall	1.93 (0.17)	174 (6)	10.0 (0.8)	219 (8)	29–69	41–55
	Box 1 (A/B)	1.78 (0.04)	174 (10)	10.7 (0.8)		A: 47 (2) B: 30 (1)	A: 48 (2) B: 43 (2)
	Box 2 (A/B)	2.10 (0.14)	176 (3)	9.3 (0.5)		A: 66 (3) B: 42 (3)	A: 54 (1) B: 50 (2)
KN95-FN2	Overall	2.59 (0.32)	219 (14)	9.4 (0.9)	282 (49)	25–37	30–39
	A	2.59 (0.33)	220 (16)	9.4 (0.9)		33 (4)	36 (3)
	B	2.58 (0.34)	217 (13)	9.3 (1.0)		28 (3)	34 (4)
KN95-ZS	Overall	1.78 (0.10)	205 (4)	12.7 (0.8)	289 (11)	78–128	36–54
	A	1.80 (0.08)	204 (4)	12.5 (0.7)		86 (8)	45 (3)
	B	1.76 (0.11)	205 (4)	12.9 (0.9)		107 (21)	45 (9)
P2-SD	Overall	2.85 (0.22)	233 (5)	9.0 (0.7)	209 (6)	64–83	53–63
	A	2.82 (0.20)	231 (4)	9.1 (0.5)	207 (5)	70 (6)	58 (5)
	B	2.91 (0.26)	236 (4)	9.0 (1.0)	214 (6)	76 (7)	57 (4)

**Table 3:** Normalized filtration performance indicators—definitions and typical ranges.

Performance Indicator	Unit	Formula	Typical Range for Charge State	
			Discharged	Charged
Normalized Penetration P_n0.3_	(−)	$P_{n0.3}=\frac{\ln\left(P_{0.3,treated}\right)}{\ln\left(P_{0.3,untreated}\right)}$	0.1–0.4	0.9–1.1
Normalized Quality Factor Q_xn0.3_	(−)	$Q_{xn0.3}=\frac{Q_{x0.3,treated}}{Q_{x0.3,untreated}}$	0.1–0.4	0.9–1.1

**Table 4:** Saturated alcohol vapor treatment and test parameters.

Test Parameter	Values	or Range		Units
Test type	Whole-mask	Flat-sheet	Flat-sheet	
Duration of exposure	5 (high)	1 (medium)	0.1 (low)	Hours
Post-treatment desorption (fume hood)	30	30	30	Minutes
Alcohol types		Ethanol Isopropanol	(EtOH) (IPA)	
Treatment ambient temperature	25 ± 0.5	25 ± 0.5	25 ± 0.5	°C
Circular sample outer/active diameter	-	109/89	109/89	mm
Filter test airflow rate through sample	64	55	55	L/min
Filter test face velocity	-	0.15	0.15	m/s
Number of samples tested	5	3	3	

**Table 5:** Test procedure settings for gel hand sanitizer exposure.

Exposure Level	Exposure Duration	No. of Releases	Release Period	Hand Rubbing	Total Mass Dispensed
	(min)	(-)	(s)	(s)	(g)
1	2	10	12	5–8	7.8
2	60	302	12	5–8	240
3	120	604	12	5–8	470
4	240	720	20	5–8	560

**Table 6:** Test procedure settings for exposure to manual spray-sanitization.

Exposure Level	No of Pulls per Spray	Release Period	Wiping	Overall Duration	No of Sprays Overall	Total Mass Dispensed
	(-)	(s)	(s)	(min)		(g)
1	3	30	10	120	240	360
2	3	30	10	240	480	720

**Table 7:** Means and one-sided 95% confidence intervals for penetration of a randomly selected mask under the combined model.

Treatment	Expected Penetration	One-Sided 95% Confidence Interval
Before treatment	0.0010	[0, 0.011)
After hand sanitizer treatment	0.0013	[0, 0.014)
After spray sanitizing treatment	0.0010	[0, 0.011)
After mask sanitization treatment	0.0031	[0, 0.017)
After saturated IPA vapor treatment	0.56	[0, 0.85)

## Data Availability

The data presented in this study are openly available from the CSIRO Data Access Portal (DAP) at https://doi.org/10.25919/0ndh-rg69.

## Supplementary Materials

The following supporting information can be downloaded at: https://www.mdpi.com/article/10.3390/ijerph19020641/s1, Figure S1: Filter test equipment used for ‘flat-sheet screening tests’; Figure S2: Filter test equipment used for ‘whole-mask screening’ tests, showing on the right the interior of the 15L test chamber; Figure S3: Filter test chamber used for ‘whole-mask MPPS’ testing with sampling provisions for pressure drop and particle concentration measurement up- and down-stream of the mask sample (left), as well as mounting provisions inside the test chamber (right); Figure S4: Normalized Penetrations P_n0.3_ from flat-sheet screening tests of KN95 masks exposed to alcohol vapors for 6 min (left, KN95-F1) or 60 min (bottom, KN95-F2). Two different post-treatments were applied: (1) ‘ventilated’ in a fume hood, (2) ‘non-ventilated’ without fume hood treatment.; Figure S5: Normalized Penetrations P_n_ from flat-sheet screening tests, showing penetrations for proxy-bacterial particles P_n2.0_ from masks (KN95-F2) exposed to saturated alcoholic vapors for 60 min.
