# Supplementary material for: The Effect of Sanitizing Treatments on Respirator Filtration Performance
Source: Int J Environ Res Public Health. 2022 Jan 6;19(2):641. doi: 10.3390/ijerph19020641 (PMC8776180; doi:10.3390/ijerph19020641)
Supplement: Supplementary file 1 [file ijerph-19-00641-s001.zip › ijerph-1446310-supplementary.pdf]

Supplementary Information: The Effect of Sanitizing Treatments on Respirator Filtration Performance

Jürg A. Schütz, Anthony P. Pierlot and David L.J. Alexander

S.1 Filter Test Equipment

The filtration performance of respirator masks and flat-sheet mask pieces was assessed by two different types of filter test equipment.

S.1.1 Particle Counter Filter Test Instrument (PCFTI)

Flat-sheet mask pieces were subjected to non-destructive 'screening tests' using the equipment shown in Figure S1. The aerosol flowrate can be adjusted from about 15 – 150 L/min and the typical aerosol concentration range is 0.01 – 0.70 mg/m^3 for this instrument. For each piece 5-8 measurements were made and averaged.

Particle concentrations were measured simultaneously up- and down-stream of the flat-sheet sample by means of two clean-room particle counters/sizers (TSI 9306-03) with 6 particle size bins. Filtration performances were calculated from results of the finest size bin ranging from 0.3 – 0.5 µm.

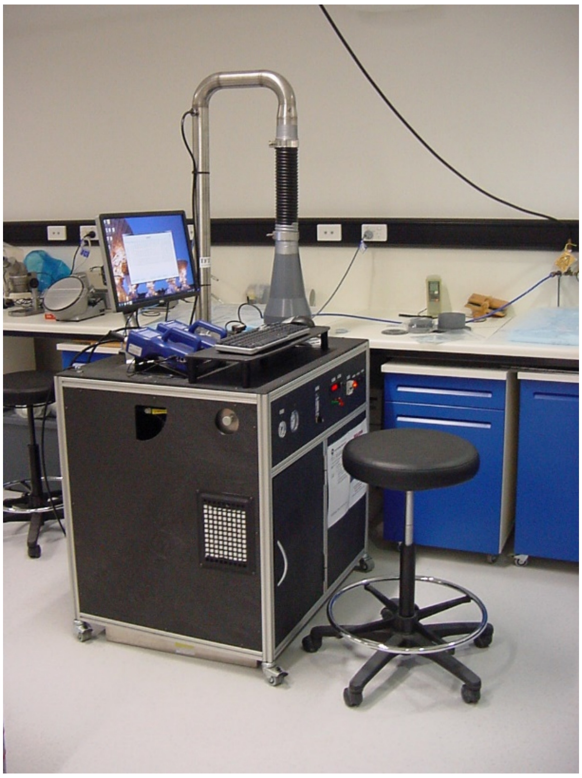

Figure S1. Filter test equipment used for 'flat-sheet screening tests'.

The pressure drop across the respirator mask or filter sample under test was measured using a Digital Manometer (TSI, Dp-Calc model 5825).

S.1.2 Hazardous Aerosol Test Duct (Test Duct)

'Whole-mask screening' and 'whole-mask MPPS' testing of respirator masks was conducted using a modular setup. The aerosol flowrate can be adjusted from 15 L/min to several thousand L/min using different configuration options.

The 'whole-mask screening' test setup shown in Figure S2 was configured with a large stainless-steel exposure chamber (15 Litres) for the masks with provisions to measure particle concentrations up- and down-stream of the mask as well as pressure drop. The solid aerosol challenge (NaCl or KCl) can be varied in its concentration range from 0.01 to about 0.60 mg/m^3 and accurately measured using a range of different particle detectors. Each measurement was an average of 6-8 individual readings and a minimum of two mask samples were tested at each exposure level.

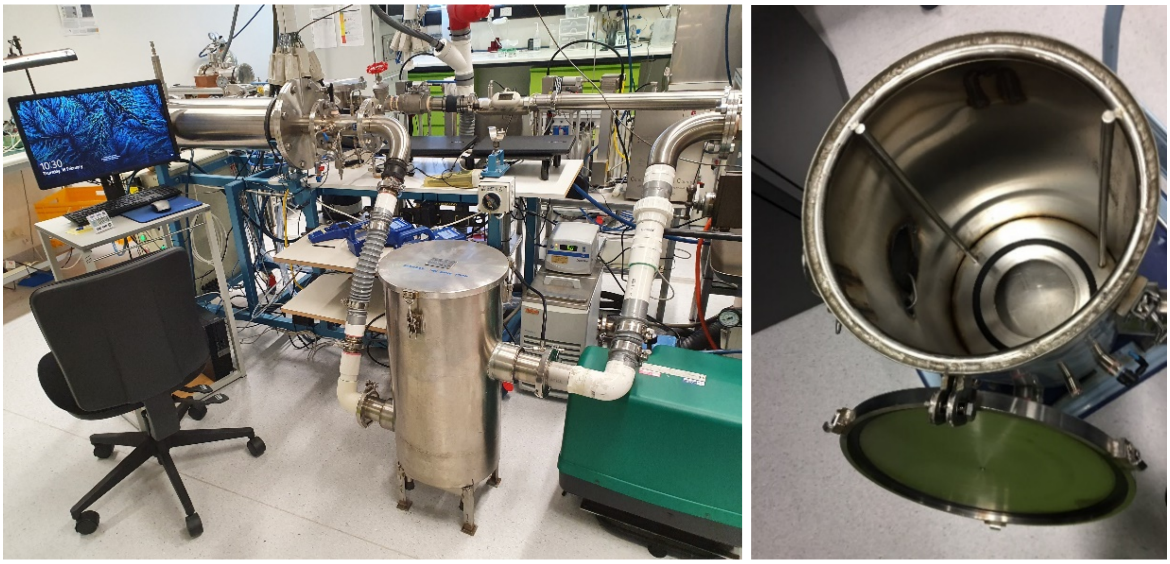

Figure S2. Filter test equipment used for 'whole-mask screening' tests, showing on the right the interior of the 15L test chamber.

Particle concentrations were measured simultaneously up- and down-stream of the flat-sheet sample by means of two clean-room particle counters/sizers (TSI 9306-03) with 6 particle size bins. Filtration performances were calculated from results of the finest size bin ranging from 0.3 – 0.5 µm.

The 'whole-mask MPPS' test differs from the 'whole-mask screening' test by the experimental chamber used, which is shown in Figure S3. The chamber is connected on both sides to provisions for particle concentration measurement, up- and down-stream of the mask, as well as for pressure drop measurement. The solid aerosol challenge (NaCl or KCl) can be varied in its concentration range from 0.01 to about 100 mg/m^3 and accurately measured using particle diluters (if required) interfaced to a range of different particle detectors. Each measurement consisted of an average of 6-8 individual readings, with a minimum of two mask samples being tested at each exposure level.

Particle detectors used for 'whole-mask MPPS' testing were:

- Optical Particle Spectrometer (OPS) TSI model 3330, with a 1:10 diluter TSI 3332-10 installed upstream for elevated particle concentrations (10 mg/m^3), used to detect particles in a size range of 0.3 – 10.0 µm
- Scanning Mobility Particle Size (SMPS) spectrometer Grimm 5.416 for particles in a size range of 0.01 – 1.00 µm

The pressure drop across the respirator mask or filter sample under test was measured using a Digital Manometer (TSI, Dp-Calc model 5825).

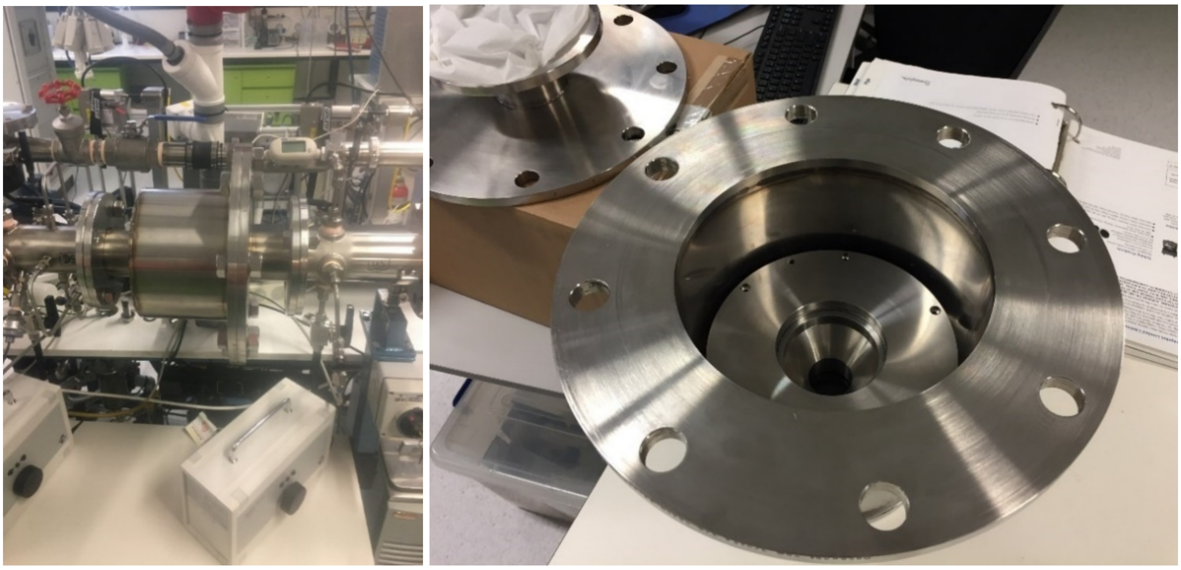

Figure S3. Filter test chamber used for 'whole-mask MPPS' testing with sampling provisions for pressure drop and particle concentration measurement up- and down-stream of the mask sample (left), as well as mounting provisions inside the test chamber (right).

S.2 Exploratory experiments with saturated alcohol vapors

Other dependencies of the effects of saturated alcohol vapors on the respirator masks were investigated via exploratory experiments using 'flat-sheet screening' filtration tests.

S.2.1 Effect of Alcohol Residues on Filter Media

Results in Figure S4 illustrate the impact of giving the filter material time to release adsorbed alcohol inside a fume hood before testing ('ventilated' state), as opposed to testing the filter material shortly after taking it out of the treatment chamber ('not ventilated' state). The desorption post-treatment was carried out immediately after exposure by placing the filter sample flat on a paper towel inside a fume hood with laminar air flow for a period of 30 minutes, according to details in Table 4 of the main text.

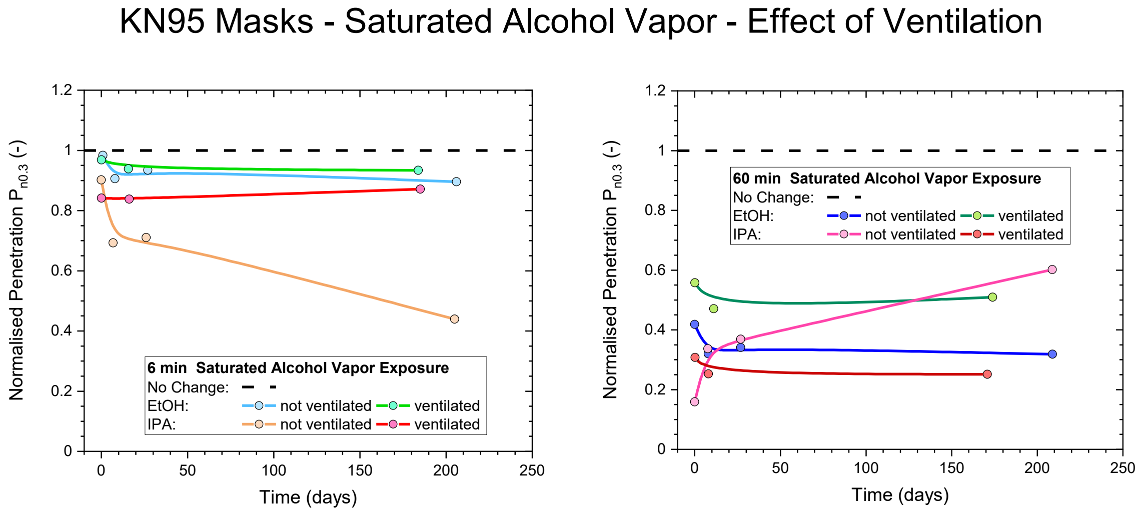

Figure S4. Normalized Penetrations $P_{n0.3}$ from flat-sheet screening tests of KN95 masks exposed to alcohol vapors for 6 minutes (left, KN95-F1) or 60 minutes (bottom, KN95-F2). Two different post-treatments were applied: 1) 'ventilated' in a fume hood, 2) 'non-ventilated' without fume hood treatment. Samples were stored in plastic bags between filter tests. Lines are provided to emphasize trends across measurements.

It appears that the desorption in the fume hood leads to more stable values for the time following the exposure with $P_{n0.3}$ values remaining constant from 3 days after treatment for all four masks that were tested. In general, EtOH produces a smaller (or equal) amount of deterioration in comparison to IPA.

The initial value of Normalized Penetration is not always followed by further decay, as noticed for the 60-minute IPA treatment without desorption, where $P_{n0.3}$ increases from 0.16 immediately after treatment to 0.34 about seven days later and to 0.6 after 7 months. This is not uncommon for filter materials where stored electrostatic charge has been significantly affected by the treatment. It appears that the electrostatic charge distribution initially becomes disrupted by the exposure and subsequently settles into a different state, which allows the filter medium to reclaim some of the filtration performance it had lost.

S.2.2 Respirable and Proxy-Bacterial Particle Size

It is interesting to note how much less the impact of alcohol exposure is for larger particles in the typical size range of aerosolized bacteria. The term 'proxy-bacterial' is used to distinguish solid particle filter testing from actual bacterial filter testing, whereby filter media are challenged with bacteria contained within liquid aerosol particles [50,51]. It is well known for particles >1 µm (brown curve in Figure 1 of main text) that the larger the particle size the less the resulting impact from an exposure to EtOH or IPA vapor, because these particles are collected physically, i.e. by the fibers of the medium, and electrostatic charge is much less important.

The 60-minute exposure plots for 'proxy-bacterial' particles (2 – 5 µm size range) are shown in Figure S5 for comparison with respirable particles (0.3 – 0.5 µm size range) shown in Figure S4.

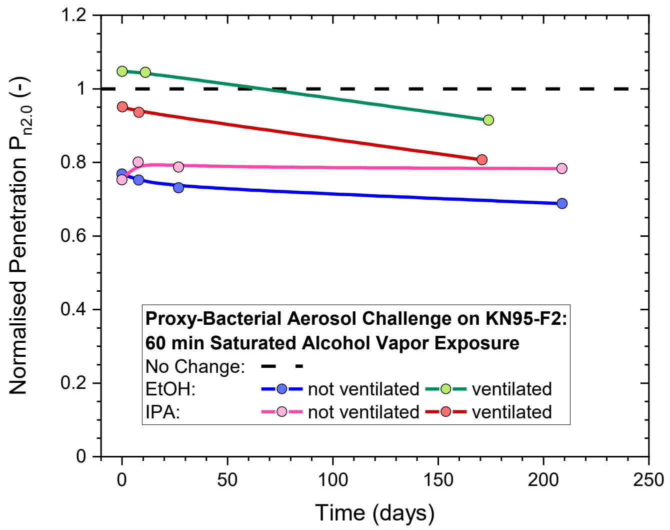

Figure S5. Normalized Penetrations P_n from flat-sheet screening tests, showing penetrations for proxy-bacterial particles $P_{n2.0}$ from masks (KN95-F2) exposed to saturated alcoholic vapors for 60 minutes. Lines are provided to emphasize trends across measurements.

The effect from desorbing alcohol residues from the mask materials is even more obvious in the proxy-bacterial particle size range with all desorbed $P_{n2.0}$ results ranging above 0.8

S.3 References

50. CEN. EN 14683:2014 Medical face masks - Requirements and test methods. **2014**, 23.
51. ASTM_International. F2101-14 Standard Test Method for Evaluating the Bacterial Filtration Efficiency (BFE) of Medical Face Mask Materials, Using a Biological Aerosol of *Staphylococcus aureus* **2014**, *F 2101 - 14*, 6.
